# The Weight of a Guilty Conscience: Subjective Body Weight as an Embodiment of Guilt

**DOI:** 10.1371/journal.pone.0069546

**Published:** 2013-07-31

**Authors:** Martin V. Day, D. Ramona Bobocel

**Affiliations:** 1 Department of Psychology, Princeton University, Princeton, New Jersey, United States of America; 2 Department of Psychology, University of Waterloo, Waterloo, Ontario, Canada; Royal Holloway, University of London, United Kingdom

## Abstract

Guilt is an important social and moral emotion. In addition to feeling unpleasant, guilt is metaphorically described as a “weight on one's conscience.” Evidence from the field of embodied cognition suggests that abstract metaphors may be grounded in bodily experiences, but no prior research has examined the embodiment of guilt. Across four studies we examine whether i) unethical acts increase subjective experiences of weight, ii) feelings of guilt explain this effect, and iii) whether there are consequences of the weight of guilt. Studies 1–3 demonstrated that unethical acts led to more subjective body weight compared to control conditions. Studies 2 and 3 indicated that heightened feelings of guilt mediated the effect, whereas other negative emotions did not. Study 4 demonstrated a perceptual consequence. Specifically, an induction of guilt affected the perceived effort necessary to complete tasks that were physical in nature, compared to minimally physical tasks.

## Introduction

In everyday language, guilt is treated as a tangible substance—people bring guilt upon themselves, carry it, or are weighed down by it. Similarly, feelings of guilt can be expressed as a “weight on one's conscience.” Such metaphoric language suggests that guilt has properties similar to an object with real weight [1]. On the one hand, weight-related adjectives may merely represent traditional descriptions of guilt. On the other hand, guilt is a real emotion, and the heaviness of guilt may be embodied as a feeling of weight. In this paper, we tested whether the experience of guilt is grounded in sensations of increased weight.

Guilt is a negative emotion that involves an awareness of responsibility for an event [2], [3]. In particular, guilt arises from a focus on a specific action, or non-action, that violates societal or personal standards [4], [5], [6]. One reason that guilt is important is because of its role in moral and social functioning [7], [8]. The anticipation of feeling guilty in the future may help prevent individuals from participating in immoral acts that violate internalized standards [9], [10]. For example, those with a stronger tendency to feel guilty are less likely to lie or act dishonestly [10], [11]. Feeling guilt following a wrongdoing can also be socially adaptive. For instance, guilt is commonly linked with reparative behaviors, such as taking responsibility, apologizing, and putting in additional effort with others [7], [11], [12].

Individuals tend to have a remarkable capacity to feel guilty. Guilt can be evoked by doing something “bad” interpersonally [4], or for private misdeeds [13], [14]. People can experience anticipated guilt for the responsibility of future actions [15], vicarious guilt for the wrongdoing of close others [16], and collective guilt for harms committed by one's ingroup [17]. In phenomenological reports, guilt is characterized not only as feeling badly, but also by feelings of tension and regret [10]. However, to our knowledge there has been no empirical examination of the subjective weight induced by guilt.

Although guilt and weight are seemingly unrelated, there is mounting evidence that cognitions are grounded in sensations and actions of the body [18], [19], [20]. For instance, holding a warm coffee cup led a target person to be rated as more interpersonally warm [21], and squeezing a soft (i.e., tender) ball led sex-ambiguous faces to be more often categorized as female [22]. In addition, recalling personal experiences of social exclusion increased reports of feeling cold [23], and reminders of immoral (i.e., dirty) acts bolstered motivations to cleanse [24], [25].

The “weight of guilt” metaphor may also reveal core links between emotional reaction to wrongdoing and sensations of weight consistent with an embodied theory of emotion [26]. Under this approach, embodiment can facilitate affective experience. The reverse pattern can also occur: emotional experience, through novel activation or recall, can facilitate embodiment [27]. Given the important role of guilt in personal and social functioning, we sought to broaden our understanding of guilt by examining embodiment.

We conducted four studies of the embodied nature of guilt. In Studies 1–3 we examine the effect of unethical acts on subjective body weight. As unethical acts can lead to feelings of guilt for violating internalized standards, in Studies 2 and 3 we also test whether guilt can account for any increase in subjective weight. As we explain in greater detail after Study 3, we then examine a possible consequence of this phenomenon (Study 4). Our guiding hypothesis in Studies 1–3 was that immoral acts, which can imbue guilt, would also lead to feelings of additional weight on the body compared to control conditions.

## Study 1

### Method

#### Participants

One hundred and fifty three Canadian undergraduates (60.1% women, 0.7% undisclosed; *M_age_* = 20.75, *SD* = 4.41) participated in exchange for course credit. Ethnic groups included 39.2% White, 28.7% Asian, 14.4% East Indian, 4.6% Middle Eastern, 1.3% Black, 0.7% Hispanic, 0.7% Aboriginal, 9.1% Other and 1.3% undisclosed. This study and the remaining studies were approved by the ethics committee of the University of Waterloo, and all participants indicated written consent.

#### Procedure

Participants were informed that they would complete two tasks to help develop materials for future research: one task concerned the description of memories and a separate task involved perceptual judgment. First, participants were randomly assigned to experimental condition. Two thirds were assigned to one of two memory conditions, whereas the remaining third was assigned to a no-memory, control condition. Next, participants in the memory conditions were asked to recall and describe in detail a time they either did something ethical or unethical, similar to past research [25]. The unethical memory condition can induce strong feelings of guilt as participants focus on a wrongful behavior from their past.

Following the manipulation, participants made a perceptual judgment, which was our measure of subjective weight. They were told that sometimes people feel more or less weight, and “Compared to your average weight, how much do you feel you weigh right now?” (1 = *much less than my average*, 6 = *exactly my average*, 11 = *much more than my average*). Participants in the control condition received the same cover story, but completed the perception task first, and were debriefed before completing the memory task. Participants' physical weight in pounds was reported in an unrelated testing session prior to study registration.

### Results and Discussion

**Figure 1:**
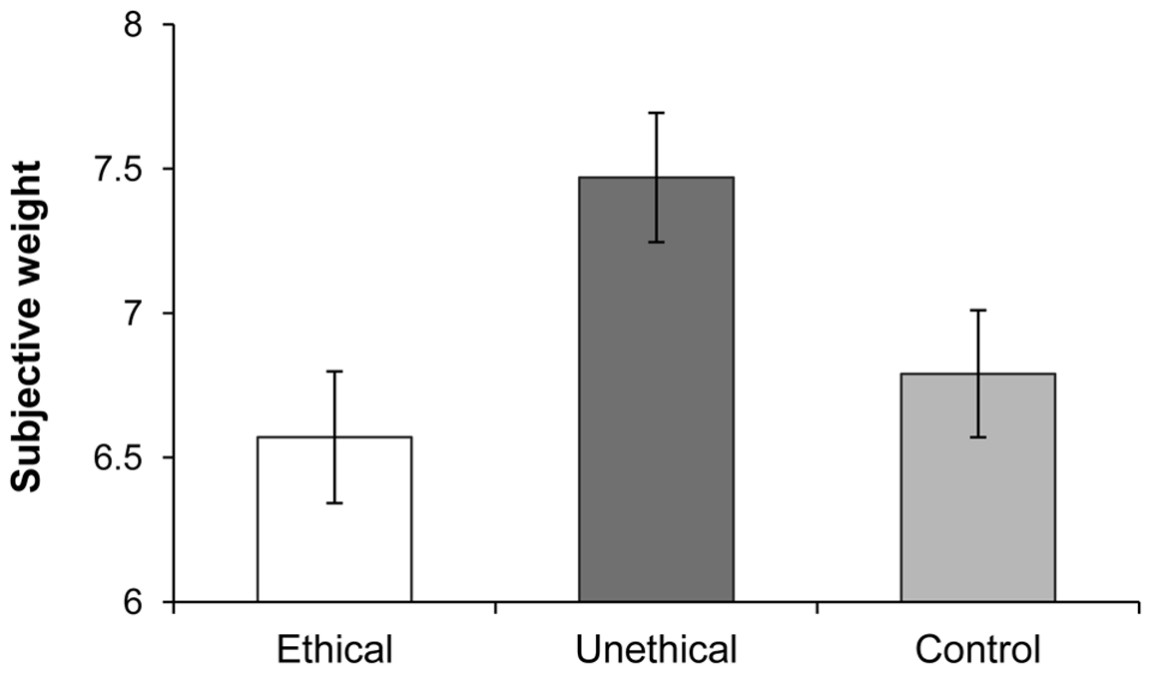
Mean ratings of subjective weight following recall of ethical or unethical events, or no recall. Study 1. Error bars indicate standard errors.

As predicted, a significant one-way ANOVA indicated that subjective weight varied by condition, *F*(2, 150) = 4.33, *p* = .02. As seen in Figure 1, contrasts revealed that compared to their average weight, participants in the unethical condition reported weighing significantly more (*M* = 7.47, *SD* = 1.63) than those in the ethical condition (*M* = 6.57, *SD* = 1.63), *t*(98) = 2.76, *p* = .007, and also more than control participants (*M* = 6.79, *SD* = 1.54), *t*(102) = 2.18, *p* = .03. There were no differences in subjective weight between the ethical and control conditions, *t*(100) = 0.70, *p* = .48. Moreover, participants' physical weight did not vary by condition, *F*<1, *ns*, and controlling for this factor did not affect the significance of the results.

Thus, when participants recalled an unethical memory, they reported higher than average subjective body weight compared to participants who recalled an ethical memory or did not recall a memory. Notably, these findings remained strong regardless of participants' physical weight. As people sometimes mention feeling lighter or elevated after performing good deeds, this figurative language might lead one to predict that ethical acts could lighten one's perception of weight. However, we found that thinking of such actions did not assuage the typical sensation of body weight compared to a neutral condition.

Study 1 provides the first evidence that violations of ethical standards may be embodied as sensations of body weight. We believe that feelings of guilt may be one factor that drives the effect. Thus, we conducted Studies 2 and 3 to replicate the results of Study 1 and examine whether feelings of guilt can explain the findings. We also sought to rule out other explanations. In Study 2 we examine whether increases in weight perceptions are the result of exposure to personal unethical acts or unethical acts in general. It is conceivable that thinking about any negative and immoral deed may “weigh on one's conscience,” and affect weight sensations. Alternatively, if guilt is related to perceptions of weight, then unethical actions irrelevant to the self should not induce greater perceptions of weight. To learn more about the characteristics and impact of the memories recalled, we also assessed common moral emotions (i.e., disgust, pride) and relevant factors of the events recalled (e.g., responsibility).

In Study 2 we again hypothesized that unethical acts will lead to perceptions of greater weight than control conditions. Moreover, unethical acts should induce feelings of guilt, which will explain reports of subjective weight.

## Study 2

### Method

#### Participants

Three hundred and eighteen U.S. participants (62.6% women, 0.9% undisclosed; *M_age_* = 33.01, *SD* = 12.04) were recruited through Mechanical Turk [28]. Ethnic groups included 79.2% White, 8.8% Black, 5.0% Hispanic, 2.6% East Asian, 0.3% East Indian, 0.3% Native American, 2.2% Other, and 1.6% undisclosed.

#### Procedure

As in Study 1, participants were randomly assigned to one of three conditions. All participants first completed a memory task. Beyond the ethical and unethical memory conditions, we added a condition in which participants recalled a time another person did something unethical. As people can feel guilt for close others' actions [16], participants described an unethical act committed by someone in the media (e.g., a celebrity, politician, sports player), who presumably is not in their social sphere. To examine whether the effect of Study 1 extends to recent acts, all participants described their most recent memory.

After describing one of the three memories, participants indicated perceptions of their body weight compared to their average weight, similar to Study 1. Participants also completed questions about the memory they described, including how much it was negative, personally important, and their degree of personal responsibility. They also reported how much the content of the memory led them to feel guilt, disgust, and pride. Guilt was measured to test for its mediating role. The other variables were included to learn more about the characteristics and impact of the memories recalled, and to examine the roles of these factors in our main results. All questions were on 9-point scales with greater numbers indicating more endorsement (e.g., more weight, emotion, etc.).

### Results and Discussion

We conducted one-way ANOVAs testing for mean differences on the dependent variables. All of these overall tests were significant, thus we also conducted contrasts between conditions (see Table 1 for means and tests of significance). As predicted, participants who recalled an unethical act reported significantly more weight compared to those who recalled an ethical memory or an unethical memory of a distant other person. The mean levels of importance, negativity, disgust or pride did not mimic the pattern found across conditions for subjective weight. Not surprisingly, therefore, controlling for each of these variables did not affect the significance of our main finding. As seen in Table 1, ratings of personal responsibility were highest in the unethical condition. Critically, however, the between condition differences in subjective weight remained significant, even when controlling for responsibility.

**Table 1:** Descriptive statistics and significance tests in Study 2.

	Ethical		Unethical		Unethical-Other			
Variable	*M*	*SD*	*M*	*SD*	*M*	*SD*	*F*	*p-value*
Weight	5.78^a^	1.37	6.35^b^	1.56	5.88^a^	1.40	4.55	.01
Guilt	1.93^a^	1.91	6.10^b^	2.34	1.63^a^	1.53	168.21	<.001
Disgust	2.21^a^	2.20	4.93^b^	2.73	6.65^c^	2.36	91.33	<.001
Pride	6.42^a^	2.34	2.10^b^	1.71	1.88^b^	1.73	180.42	<.001
Negative	3.61^a^	2.23	6.52^b^	1.62	6.96^b^	1.60	103.72	<.001
Importance	5.49^a^	2.36	5.07^a^	2.35	3.35^b^	2.37	25.15	<.001
Responsibility	6.51^a^	2.80	7.62^b^	2.15	1.26^c^	1.14	79.30	<.001

Note. Different superscripts within rows indicate means that differ significantly, *p*<.05.

**Figure 2:**
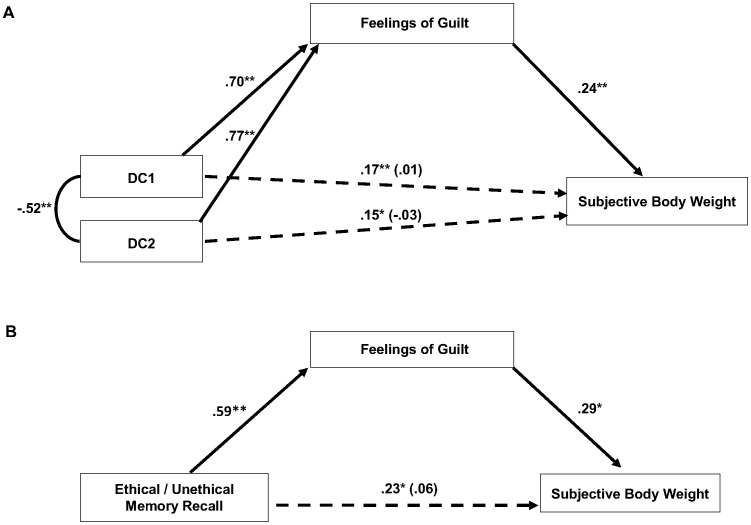
Mediation models in Studies 2 and 3. Study 1. These models examine the role of feelings of guilt in the relation between the memory manipulation and subjective perceptions of weight. Model A (Study 2) depicts the three experimental conditions dummy-coded as two variables. For DC1 the Ethical condition is coded as -1 (0, 0, -1) and for DC2 the Unethical-Other condition is coded as -1 (0, -1, 0). Model B (Study 3) displays the Unethical (1) and Ethical (0) conditions. Coefficients are standardized betas. Numbers in brackets are associations in the final model controlling for the mediator. A, N  =  311; B, N  =  91; * p < .05, ** p < .01.

Next, we examined whether increases in subjective weight can be explained by feelings of guilt following recall of unethical acts, as compared to the two control conditions (i.e., ethical, unethical-other). The zero-order correlations of the relevant variables can be seen in Table 2. For mediation analyses we created two dummy-coded variables (DC1, DC2) to account for the three conditions. For DC1, the ethical condition was coded as −1 compared to the other conditions (0, 0, −1), and for DC2, the unethical-other condition was coded as −1 (0, −1, 0). The unethical condition was coded as 0 in each case. We used Structural Equation Modeling (SEM) to examine associations among dummy-coded variables, guilt, and subjective weight (see Figure 2A). To fully represent our findings we report the unstandardized results in text and the standardized results in the figure. First, we demonstrated that the memory manipulation affected the dependent variable. As compared to control conditions, the regressions revealed that recalling an unethical act led to increased subjective weight (DC1: *b* = 0.54, *SE* = .20, *p* = .008; DC2: *b* = 0.45, *SE* = .20, *p* = .03). Next, we added guilt to the model (mean-centered). The manipulation also led to increased feelings of guilt (DC1: *b* = 4.17, *SE* = .27, *p*<.001; DC2: *b* = 4.48, *SE* = .27, *p*<.001). When the manipulation variables and guilt scores were simultaneously allowed to predict subjective weight, the association between the memory manipulation and subjective weight was reduced (DC1: *b* = 0.03, *SE* = .27, *p* = .91; DC2: *b* = −0.10, *SE* = .27, *p* = .70). Consistent with mediation, the association between guilt and subjective weight remained significant (*b* = 0.12, *SE* = .04, *p* = .003). To test mediation statistically, we followed bootstrapping procedures using 3000 resamples [29]. The indirect paths of guilt were significant (DC1: *b* = 0.51, *SE* = .18, DC2: *b* = 0.55, *SE* = .20) as indicated by bias-corrected 95% Confidence Intervals (CI) that were different from zero (DC1: 0.17, 0.90; DC2: 0.19, 0.97). The results of the preceding tests therefore support the hypothesis that the experience of guilt can explain how the unethical memory manipulation led to increased subjective weight.

**Table 2:** Correlations among predictor and dependent variables in Study 2.

Variable	1	2	3	4	5	6
1. DC1	(-)	−.52**	.30**	.56**	−.73**	.10
2. DC2		(-)	.40**	−.49**	.41**	.06
3. Guilt			(-)	.26**	−.30**	.22**
4. Disgust				(-)	−.53**	.09
5. Pride					(-)	−.10
6. Subjective weight						(-)

The DC1 (0, 0, −1) and DC2 (0, −1, 0) dummy-coded conditions use the Unethical condition as a reference (coded as 0 in both cases). Note.

* *p*<.05,

** *p*<.01.

Whereas we had theoretical reason to predict that feelings of guilt can explain the effects of our manipulation on subjective weight, we did not have a clear theoretical basis to expect the same for the other emotions we assessed (disgust, pride). Nevertheless, we remained open to the possibility that the other emotions might explain reports of subjective weight. For instance, it is conceivable that increased feelings of disgust following unethical acts contributed to increased perceptions of weight. It is also possible that a loss of pride or sinking feeling, increased subjective heaviness. Therefore, we explored the possible mediating role of disgust and pride. To follow up, we used the same procedures as for guilt, to separately examine whether disgust and pride mediated the effect of our manipulation on perceptions of weight. As evident by the non-significant indirect paths of disgust (DC1: *b* = 0.13, *SE* = .11, CI [−0.07, 0.36]; DC2: *b* = −0.08, *SE* = .07; CI [−0.23, 0.04]), and pride (DC1: *b* = 0.14, *SE* = .20, CI [−0.22, 0.56]; DC2: *b* = −0.01, *SE* = .02; CI [−0.08, 0.01]), these emotions did not show evidence of mediation.

Taking our findings in Study 2 together, as predicted we found that the recall of recent unethical actions led to greater estimates of subjective weight than did recall of either ethical deeds or unethical actions of distant others. Moreover, participants' reports of additional weight were consistent with their feelings of guilt for their unethical actions. Other emotions, including disgust and pride did not explain the subjective weight results, nor did judgments of importance, responsibility, and event negativity.

Although Study 2 replicated Study 1 and provided a demonstration of mechanism, it is worthwhile to replicate this effect and explore other explanations. For example, in Study 3 we test whether sadness, a common emotion related to negative experiences, is uniquely related to sensations of weight. In addition, we examine how specific the effect is to weight, or whether other perceptual estimates (e.g., tallness) are also affected. Doing so enables us to examine the discriminant validity of our findings on subjective weight. It would be less consistent with our weight of guilt hypothesis if our manipulation affected many non-weight related body judgments (e.g., tallness), which, if found, could suggest the occurrence of broader processes or bias in participant responding.

## Study 3

### Method

#### Participants

Ninety three U.S. participants (41.9% women; *M_age_* = 28.58, *SD* = 8.88) were recruited for an online study as in Study 2. Ethnic groups included 73.1% White, 11.8% Black, 11.8% Asian, 1.1% Hispanic, and 2.2% Other.

#### Procedure

The manipulation in Study 3 was similar to Study 2 except that we focused on two memory conditions, ethical and unethical, again with random assignment of participants to condition. We also examined whether the same effect could be found when reducing the amount of recalled content; thus we asked participants for only a concise description of their ethical or unethical deed. After providing their memory, participants answered a series of questions about how they physically perceived themselves. In Studies 1 and 2 we used a single-item measure of subjective weight. In an effort to increase the reliability of our dependent measure, we added a conceptually similar item [30]. Participants first reported their subjective heaviness, followed by their subjective weight. We created an index of subjective weight by combining these two items (*r* = .46, *p*<.001). To examine if our manipulation affected other body-related estimates, participants also reported their subjective tallness, ability to hear and smell, and subjective age. Participants were asked the same memory related questions as in Study 2. Particular to this study, participants also indicated feelings of sadness and excitement. All questions were on 9-point scales, with higher numbers indicating more endorsement (e.g., more height, ability, etc.).

### Results and Discussion

We conducted one-way ANOVAs testing for mean differences on all questions (see Table 3). Once again, thinking about an unethical deed led participants to report greater subjective weight compared to the control condition. If the same manipulation also significantly affected several other perceptual estimates then such findings would weaken support for our hypothesis that unethical acts are linked to subjective perceptions of weight in particular. However, the other perceptual estimates (tallness, hearing ability, sense of smell, subjective age) did not vary between conditions, thus strengthening support for our hypothesis (for results of these tests see Table 3).

**Table 3:** Descriptive statistics and significance tests in Study 3.

	Ethical		Unethical			
Variable	*M*	*SD*	*M*	*SD*	*F*	*p-value*
Subjective perception						
Weight	4.54	1.48	5.28	1.57	5.46	.02
Tallness	5.02	1.11	4.78	1.24	0.96	.33
Hearing	5.40	1.56	5.33	1.42	0.06	.80
Smell	5.33	1.40	5.29	1.36	0.01	.91
Age	5.08	1.70	5.20	1.93	0.10	.75
Memory						
Guilt	1.80	1.51	4.90	2.76	46.43	<.001
Disgust	2.17	1.90	3.56	2.60	8.86	<.01
Pride	5.90	2.22	2.90	2.53	36.98	<.001
Sadness	2.15	1.75	3.34	2.88	6.03	.02
Excitement	4.23	2.60	3.24	2.55	3.36	.07
Negative	2.35	1.86	6.80	1.87	129.30	<.001
Responsibility	7.27	2.35	8.12	1.68	3.84	.05

As this study used a new subjective weight item, we also examined the effect of the manipulation on each weight-item. Unethical acts led participants to feel more subjective heaviness (*M* = 4.90, *SD* = 2.01) than ethical acts (*M* = 3.81, *SD* = 1.96), *F*(1, 91) = 6.99, *p* = .01. Recalling unethical acts also led to reports of more subjective weight (*M* = 5.66, *SD* = 1.61) than ethical acts (*M* = 5.22, *SD* = 1.49), however, this pattern was not significant *F*(1, 91) = 1.88, *p* = .17. It is unclear why the same pattern was found for both items, but was stronger for the heaviness item. One possibility is that there were fewer participants per condition in Study 3, compared to Studies 1 and 2, and thus the relatively low power may have contributed to this result. We did not counterbalance the presentation order of the subjective weight items, and thus an additional possibility is that participants may have tended to assuage their responses, after responding to the initial heaviness item. As the subjective weight index was composed of conceptually consistent and face-valid items that were reasonably correlated for a 2-item measure, and because the items revealed a consistent pattern of results, we employed the subjective weight index in the remaining analyses.

Participants in the unethical condition indicated the most guilt, disgust, and sadness, whereas those in the ethical condition reported the most pride and excitement. Compared to the ethical condition, the unethical condition was rated as more negative and involved more personal responsibility. However, controlling for negativity and personal responsibility did not significantly affect the results. Moreover, when we controlled for feelings of disgust, sadness, pride, and excitement, the main results remained strong, with the exception of sadness, in which case the pattern of means was the same, but the overall *p*-value was marginal, *F*(1, 90) = 3.53, *p* = .06.

We again tested whether feelings of guilt mediated the association between the memory manipulation and subjective weight (see Table 4 for zero-order correlations). Consistent with Study 2, we conducted mediation tests using SEM. As seen in Figure 2B, the unethical condition led to relatively higher ratings of subjective weight (*b* = 0.73, *SE* = .32, *p* = .02). Next, we added guilt to the model. The manipulation also predicted guilt (*b* = 3.10, *SE* = .45, *p* = .001). In turn, guilt predicted subjective weight (*b* = 0.17, *SE* = .07, *p* = .04). The association between the manipulation and subjective body weight was reduced (*b* = 0.20, *SE* = .38, *p* = .60). Bootstrapping procedures (3000 resamples) indicated that the indirect path of guilt was significant (*b* = 0.53, *SE* = .27, bias-corrected 95% CI [0.04, 1.12]). Therefore, these results support the mediating role of guilt, as in Study 2.

**Table 4:** Correlations among predictor and dependent variables in Study 3.

Variable	1	2	3	4	5	6	7
1. Unethical-Ethical	(-)	.59**	.30**	−.54**	.25*	−.17	.23*
2. Guilt		(-)	.72**	−.54**	.70**	−.23*	.33**
3. Disgust			(-)	−.37**	.72**	−.11	.21*
4. Pride				(-)	−.30**	.60**	−.13
5. Sadness					(-)	−.13	.22*
6. Excitement						(-)	−.01
7. Subjective weight							(-)

Unethical and Ethical memory conditions were coded as 1, 0, respectively. Note.

* *p*<.05,

** *p*<.01.

We also considered the possibility of whether the other emotions assessed (disgust, pride, sadness, excitement) were mediators. The correlations in Table 4 indicate that beyond guilt, only disgust and sadness were also related to the independent and dependent variables. Thus, we followed the same tests of mediation for disgust and sadness as for guilt. The nonsignificant indirect path of disgust (*b* = 0.14, *SE* = .12, CI [−0.04, 0.48]), and sadness (*b* = 0.13, *SE* = .12, CI [−0.02, 0.48]) indicated that neither of these emotions were independent mediators.

We note that in Studies 2 and 3 we examined the possible mediating roles of guilt and other emotions separately. Alternatively, it is possible to test mediators simultaneously. This procedure is most relevant when testing competing hypothesis of mediating factors [31]. Whereas we have theoretical reason to test the role of guilt, alternative explanations using other emotions are less evident. Moreover, it is quite possible that testing multiple mediation in this instance could lead to erroneous conclusions. For example, we observed generally high correlations among the emotions. This is not surprising in light of known difficulties when measuring guilt concurrently with other emotions, such as anchoring scores on guilt when measured first [10], [32]. Thus, in this instance, testing mediators simultaneously could lead to artificial attenuation of the explanatory power of individual mediators because relevant variance that is shared among the mediators is partialled out [31].

In sum, Studies 1–3 demonstrated that unethical acts that lead to feelings of guilt can be embodied as a sensation of additional weight. One implication of physically being weighed down is that it can affect judgments related to physical behaviors [33]. For instance, additional weight (e.g., by wearing a backpack) may increase perceptions of how much energy is required to complete physical tasks. To test for a possible downstream consequence, we harnessed this general notion in Study 4, conceptually replacing physical weight with the weight of guilt. As unethical acts can induce the subjective experience of additional weight on the body, we expected that the same manipulation would lead to greater perceived effort to complete physical tasks, but would not affect estimates of effort for nonphysical tasks.

## Study 4

### Method

#### Participants

Sixty seven U.S. participants (46.3% women; *M_age_* = 28.48, *SD* = 9.93) were recruited for an online study as in Studies 2 and 3. Ethnic groups included 68.7% White, 9.0% Asian, 7.4% Black, 7.4% Hispanic, 1.5% Middle Eastern, 1.5% Native American, and 4.5% Other.

#### Procedure

Participants were randomly assigned to recall either an unethical or ethical memory, as in Study 3. Afterwards, participants made perceptual judgments. To assess the perceived effort of behaviors, participants were presented with a variety of tasks and indicated how much energy and effort each task would require (1 = *Not at all*, 9 = *Very much effort and energy*), similar to past research [34]. Consistent with the tendency for guilt to be associated with reparative actions [10], [12], we framed the items as prosocial behaviors. Three questions involved physical effort (carrying groceries upstairs for someone, helping someone move, carrying a basket of laundry for someone, α = .75), and three questions involved minimal physical effort (giving someone change, holding an elevator for someone, donating online, α = .71).

### Results and Discussion

We conducted a 2 (Unethical vs. Ethical)×2 (Physical vs. Nonphysical) mixed ANOVA on perceived task effort, with repeated measures on the last variable. Not surprisingly given the manipulation, there was a strong within-subjects effect of physical tasks, *F*(1, 65) = 160.22, *p*<.001. This finding can be interpreted as a manipulation check on the physical effort items, where physical tasks were perceived to involve more effort and energy (*M* = 4.53, *SD* = 1.54) than the nonphysical tasks (*M* = 2.37, *SD* = 1.47). There was no main effect of the morality of recall, *F*<1, *ns*.

**Figure 3:**
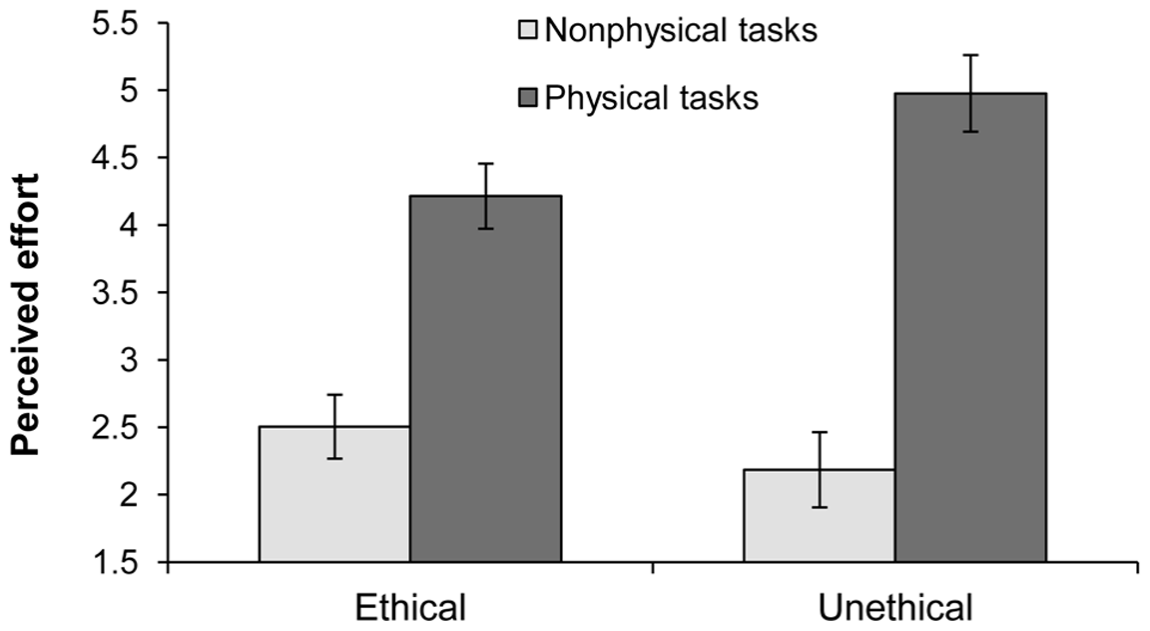
Mean perceived effort of physical and nonphysical tasks following recall of ethical or unethical events. Study 4. Error bars indicate standard errors.

Importantly, the predicted interaction was significant, *F*(1, 65) = 9.26, *p* = .003. As seen in Figure 3, the physical tasks were perceived as more effortful by those who had just recalled an unethical, weight-of-guilt inducing memory (*M* = 4.98, *SD* = 1.54) than participants who had recalled an ethical memory (*M* = 4.21, *SD* = 1.48), *F*(1, 65) = 4.18, *p* = .04. For nonphysical tasks, there was no significant difference between the unethical (*M* = 2.18, *SD* = 1.35) and ethical conditions (*M* = 2.50, *SD* = 1.56), F<1, *ns*. In other words, the same manipulation that instilled perceptions of weight related to guilt in our earlier studies was also found to affect judgments of effort for completing physical, but not nonphysical tasks. We believe this pattern of results occurred because the weight of guilt made the physical tasks appear as more effortful to complete. Whereas Studies 1–3 established that induced guilt predicted subjective body weight, Study 4 builds upon these findings by demonstrating a consequence consistent with increased weight.

We recognize that the weight of guilt is not the only mechanism that may alter physical effort perceptions. For instance, having more frequent and bothersome thoughts surrounding a kept secret is correlated with effort perceptions [34]. Nevertheless, the present study demonstrates an effect that is most consistent with the embodiment of the affective experience of guilt, and is consistent with the results of Studies 1–3.

## General Discussion

Guilt is a common emotional experience following an unethical deed. Four studies revealed how actions that imbue feelings of guilt may be embodied and can affect judgments. Extending the metaphor that guilt is a heavy weight on people's conscience, Studies 1–3 demonstrated that immoral acts led to reports of increased subjective body weight compared to control conditions. Study 1 isolated the direction of the effect: unethical acts made participants feel heavier, but ethical acts did not make participants feel lighter. Studies 2 and 3 found that increased feelings of guilt can explain greater subjective weight, rather than feelings of disgust, pride, or sadness. Finally, Study 4 demonstrated that the same manipulation affected judgments consistent with the effects of physical weight. Physically demanding behaviors were perceived as more effortful to complete following recall of unethical as compared to ethical acts, thus indicating a consequence of the weight of guilt phenomenon.

Our examination of guilt contributes to the understanding of this important moral emotion and supports an embodied emotion perspective [26]. In particular, these findings demonstrate that the emotional experience of guilt can be grounded in subjective bodily sensation. In this first demonstration, we focused on the role of guilt and its effects, rather than the bidirectionality of the weight of guilt. Future research could explore whether the simulation of guilt (e.g., through physical means) may facilitate affective experience and understanding of emotion-related content [27]. Another possibility would be to test the boundaries of the weight of guilt, such as by determining whether this embodied metaphor has effects in unrelated domains [19].

Beyond confirming our hypotheses and supporting related theory, there are concerns and limitations related to our investigation as well as potential future research directions. One concern raised in the review process was the possibility that increases in reported subjective weight were due to some participants recently eating, and thus gaining weight, prior to study participation. However, such a possibility cannot readily explain the pattern of subjective weight results observed given that participants were randomly assigned to one of the two (or three) study conditions, in all studies.

Another concern raised was whether participants reported more subjective weight because they associated unethical acts with subjective weight, and believed that indicating more weight was the “correct” answer. To help reduce the possibility of such demand characteristics, in all our studies we disguised the true study purpose by employing a cover story that conceptually separated our manipulation from our measures. Moreover, in Study 2, we asked participants to recall either unethical acts of their own, or of distant others, and found that only personal unethical acts led to increased weight. This suggests that it was not the category of unethical acts in general that led to reports of increased subjective weight. Rather, our tests of mediation imply that feelings of guilt played a role. We acknowledge that it is difficult to completely rule out the possibility that participants associated the concept of guilt with weight once they experienced guilt. In part, this is because we believe that guilt is responsible for the increased sensation of subjective weight. We did, however, strive to reduce the likelihood that participants would make a direct association between guilt and weight by requesting them to recall unethical acts instead of acts for which they feel guilty, which may have been more likely to prime the concept of guilt. Future research could examine if the semantic prime of guilt does or does not have a mediating role in the association between guilt and increased subjective weight. It may also be of interest to pinpoint mediating processes between the manipulation of unethical acts and feelings of guilt. For example, future research could examine whether body postures have a role [35], [36].

Although this research was centered on the role of guilt and the weight of guilt metaphor, it is important to consider other emotions. We tested disgust, pride, and sadness, but did not find evidence that these emotions could explain reports of additional weight in our studies. These findings are encouraging, but additional research that manipulates the experience of other emotions would help to confirm or disconfirm their association with increased weight. Still, if such associations were found, it would be important to examine whether there are overlapping mechanisms at play. For example, guilt is sometimes associated with shame. As in the present research, guilt can be evoked by focusing on actions that do not meet internalized standards (e.g., “I engaged in an unethical behavior”), whereas shame tends to be evoked by broad negative self-evaluations (e.g., “I am a terrible person”) [5], [10]. Guilt and shame are distinct emotions, but individuals sometimes confuse them. This presents problems for measuring them simultaneously in research [32], which was one reason why shame was not assessed in the present studies. In addition to difficulties in measurement, we did not suppose shame to be primarily responsible for any increased weight. Shame has been more commonly associated with feeling physically small, or a desire to hide the self [10]. Thus we suspect that the embodiment of shame may be more related to physically making the body small (e.g., by crouching). This prediction would need to be confirmed in future research.

One potential limitation of the present research is that only situational guilt was examined, but it is possible that weight related to guilt may also vary depending on individuals' propensity to experience guilt [10], [11]. Although we did vary our manipulation somewhat across studies, other variations could manipulate vicarious [16], or collective guilt [17], or examine whether the anticipation of future guilt shows similar, weaker, or stronger effects [37], [38]. Finally, in Study 4 we examined how the weight of guilt affected perceptions of effort to complete physical acts. One interpretation of this result is that the weight of guilt could function to slow individuals' exertion of physical effort, which in turn provides the opportunity for contemplation about how to repair the relevant violation. Future research could shed light on this possibility.

In conclusion, the present research revealed that personal experiences of immorality can be partly understood by sensations of weight, and that guilt appears to have some responsibility for this effect. Although guilt is literally weightless, we demonstrate that the embodiment of guilt can have consequences as if it does indeed have weight. As this was our initial investigation on this topic we hesitate to draw broad or strong conclusions based exclusively on these findings. Replications using other methodologies and examinations of complementary embodied processes related to guilt may reinforce our results. Generally, we believe that further research on this topic may lead to a broadened understanding of the nature of guilt and related downstream effects.
